# Vaping—An Emerging Threat to Youngsters of Pakistan

**DOI:** 10.1111/adb.70017

**Published:** 2025-01-21

**Authors:** Nazish Jaffar, Hafiza Tooba Siddiqui, Huda Amin, Md Ariful Haque

**Affiliations:** ^1^ Department of Pathology, Sindh Medical College Jinnah Sindh Medical University Karachi Pakistan; ^2^ Sindh Medical College Jinnah Sindh Medical University Karachi Pakistan; ^3^ Nixor College Karachi Pakistan; ^4^ Department of Public Health Atish Dipankar University of Science and Technology Dhaka Bangladesh; ^5^ Voice of Doctors Research School Dhaka Bangladesh; ^6^ Department of Orthopaedic Surgery Yan'an Hospital Affiliated to Kunming Medical University Kunming Yunnan China

**Keywords:** Vape, e‐cigarettes, Pakistan

We would like to draw your attention to a critical prevailing issue regarding the usage of e‐cigarettes and their ill effects on health. E‐cigarettes have been marketed as a safer alternative to traditional tobacco products, but research has shown that they still pose significant health risks. Studies have revealed that the chemical compounds in e‐cigarettes can cause damage to the lungs, heart and nervous system [1].

With the boom in vaping culture, many people believe that e‐cigarettes are a healthier alternative to traditional cigarettes. However, research studies have shown otherwise. Studies have shown that e‐cigarettes can be harmful to human health, particularly among teenagers whose brains and respiratory systems are still developing [2]. An interesting study was published in your esteemed journal, *Addiction Biology*, on the neural performance of abstinent smokers. It aimed to find out the alterations in the brain networks. Before and after using e‐cigarettes, this experiment revealed that the impact of e‐cigarettes could be similar to neural activity caused by traditional cigarettes and other forms, which may lead to addiction [3].

To begin with, it is essential to understand that e‐cigarettes contain nicotine, which can lead to addiction and dependency similar to regular tobacco products. Studies suggest that vaping can expose users to higher levels of nicotine than traditional smoking, as well as other harmful chemicals such as formaldehyde and acetaldehyde. These chemicals are known carcinogens and could cause long‐term harm to the respiratory system (Figure 1). This shows the average exposure to substances used in e‐cigarettes. To calculate the chemical exposure from tobacco‐related items, a study was performed where the toxicological threshold for margins of exposure (MOE) for each chemical was determined as MOE < 10 as ‘high risk’. At the same time, MOE < 100 was judged as ‘risk’. More than 100 were acceptable. In this experiment, nicotine showed (a margin of exposure) MOE < 1, making it fall in the high‐risk category [3, 4]. Therefore, increasing public awareness about the potential risks of using e‐cigarettes is crucial.

**FIGURE 1:**
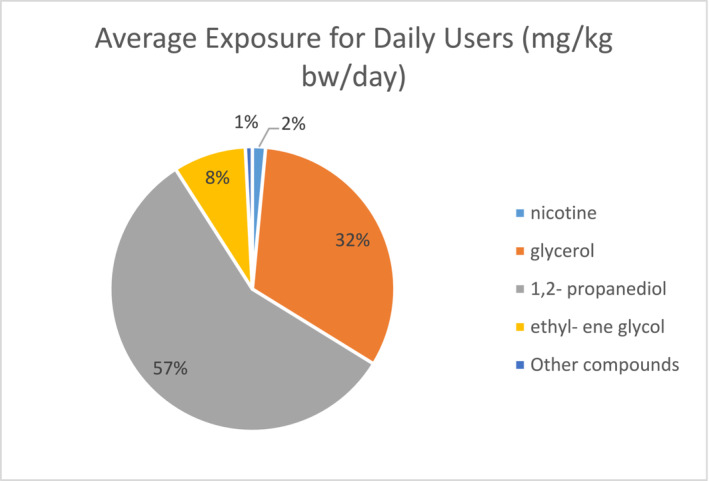
Average exposure for daily users (mg/kg/day).

Moreover, there is increasing evidence linking e‐cigarette use with cardiovascular risk factors such as high blood pressure and impaired heart function. Nicotine plays an integral role in these risks by constricting arteries and narrowing blood vessels, thereby reducing oxygen supply throughout the body and leading to damage over time [5]. As the trends in everything change, social media is a new platform where businesses market their products in the marketing industry. One of its ways is how influencers are positively marketing E‐cigarettes with no age restrictions and no trigger warning related to their adverse effects. In countries like Asia and the US, most of the followers are youngsters aged 13–17, which will ultimately expose them to the greater risk of adopting this culture. As a recent study showed, Generation Z generally perceives influencers as credible and authentic sources of information [6, 7].

Another area of concern surrounding vaping pertains to individuals who have never smoked before trying their hands at vaping. Studies have suggested that adolescents who take up e‐cigarettes are more likely to go on smoking combustible cigarettes or other forms of tobacco products later on in life than those who do not vape. E‐cigarettes are heavily promoted in Pakistan via print and electronic media as a risk‐free alternative to smoking regular cigarettes or as a tool for quitting smoking. Electronic cigarettes are sold to people of all ages in many stores and supermarkets across Pakistan's major cities, including Karachi, Lahore and Islamabad. Many tobacco businesses openly promote these new tobacco products to the young Pakistani population because there is no legislation governing e‐cigarettes [8]. According to reports, 19.1% of the Pakistani population are smokers, out of which 6.2% use e‐cigarettes, while 15.9 million (12.4%) use smokeless tobacco [9]. One of the studies showed an alarming 10.7% prevalence of smoking in teenage boys and girls aged between 13–15 years in the Pakistani population [10].

In conclusion, the risks associated with vaping are numerous and potentially harmful—from increased nicotine exposure levels to long‐term respiratory and cardiovascular risks. Therefore, raising general public awareness through educational initiatives alongside sensible policymaking will help protect both present and future generations from potential harm associated with e‐cigarette use. Such awareness campaigns could include education and cessation programs targeted towards youths and leveraging social media platforms for better outreach.

We recommend that policymakers in Pakistan must take a data‐driven approach to regulating e‐cigarettes. This would involve conducting further research studies on the effects of vaping on human health and how effectively it can prevent the onset of addiction or dependency. As part of such an approach, policymakers could also consider implementing stricter regulations on marketing practices aimed at promoting e‐cigarette usage among minors, particularly on social media marketing, as it is spreading the use of E‐cigarettes among people who were previously non‐smokers.

## Author Contributions

Nazish Jaffar provided critical insights, finalized the manuscript and performed a rigorous critical review to enhance its quality. Hafiza Tooba Siddiqui undertook the initial drafting of the letter, shaping the core message and structure. Huda Amin conceived the idea and conducted an extensive literature search, laying the foundation for the letter. Md Ariful Haque assisted in the final review process and managed correspondence with the journal, ensuring seamless communication and compliance with submission requirements.

## Ethics Statement

The authors have nothing to report.

## Consent

The authors have nothing to report.

## Conflicts of Interest

The authors declare no conflicts of interest.

## Data Availability

Data sharing is not applicable to this article as no datasets were generated or analysed during the current study.
